# The American Medical Association

**Published:** 1896-09

**Authors:** J. M. Walker


### The American Medical Association.

Reported for the DENTAL REGISTER by MRS. J. M. WALKER.

The American Dental Association held its forty-seventh annual session in Atlanta, Ga., May, 1896.

The meetings of the Dental and Oral Sections were held in the Ladies' Reception Room of the Kimball House.

DR. R. R. ANDREWS, of Cambridge, Mass., was in the chair; Dr. E. S. Talbot, Secretary.

The Section was called to order at 3 P. M., Tuesday, May 5th.

The chairman, in his address, defined the aim of the Section as being the consideration of the higher themes in the realm of scientific research—along the borders of the unknown. He said that evidence of the advancement of the dental profession was found in the growing tendency to band together in stomatological associations, having for their object the consideration and discussion of questions purely scientific. Among the more recent achievements he mentioned the work of Dr. G. V. Black. His experiments, conducted on an accurate scientific basis, have thrown much light on the physical properties of the teeth and the various filling materials in use.

Much thought is being devoted to electrical medication, especially cataphoric methods in obtunding sensitiveness of dentine, and in bleaching teeth.

In the educational field the standard of the schools are advancing, and dental graduates are taking a higher ethical and professional standing. We are now looking forward to the time when all the specialties of the healing art shall be taught in medical universities, where all shall receive a common degree.

The surgical and medical treatment of the mouth and associate parts are fully as important as those of the eye or the ear, and should be taught under the same medical auspices and accorded the same distinction.

The special topic of Professor Andrews' address was the finer processes which take place in the building up of enamel, being a review of, and friendly criticism of, the first installment of Dr. J. Leon Williams' series of papers on "The Formation and Structure of Dental Enamel," published in the *Dental Cosmos*, February, 1896.

The special point dwelt upon, in regard to which Dr. Williams and Dr. Andrews differ widely, is the function of the "stratum intermedium."

Professor Andrews commended highly the work of Dr. Williams, as a substantial contribution to the literature of the subject, and especially valuable as showing, for the first time, what the so-called *stellate reticulum* really is.

He pronounced the photo-micrographs of Dr. Williams the clearest and finest he had ever seen, but thought some of the appearances were due to post-mortem changes in the tissue, and, therefore, misleading; that it is necessary to photograph the tissue as near the life of the animal as possible.

In the brief discussion given the paper, Dr. Younger, San Francisco, expressed his approval of the views of Professor Andrews on the subject of "Dental Education."

Dr. H. D. Wilson (Bainbridge, Ga.) admired the determined tenacity with which the essayist takes hold of his subject, and regretted that he was not sufficiently well-posted to discriminate in the fine points involved.

Dr. Walker (Pass Christian, Miss.) said that in view of the statements made by the essayist as to the aims and objects of the Section, the name of the Section (Dental and Oral Surgery) was not sufficiently broad, and suggested that the name be changed to

"Section on Stomatology," thus placing it in line with the sections of Ophthalmology, Laryngology, Otology, etc.

DRS. ANDREWS and TALBOT *looked* approval of this suggestion, and at a later session Dr. Talbot announced that a resolution to that effect had been introduced in the General Session of the Association, which, however, has to lie over for one year.

On motion, the thanks of the Section were tendered Dr. Andrews for his magnificent presentation of the subject.

On motion, subject passed.

DR. S. W. FOSTER (Atlanta) read a paper entitled "Disease of the Oral Cavity a Potent Factor in General Disease." Doctor Foster spoke of the baneful sequences which follow diseases of the teeth and oral cavity, and the relationship these diseases sustain to general disease.

The oral cavity has numerous important functions—articulation and vocalization of speech—the prehension, mastication and insalivation of food, etc. From the first breath of life to the last breath of old age, the functions of the organs of the oral cavity are pre-eminently essential to life and health, and any pathological condition here is immediately made manifest in other organs of the body, often the most remote. It is an admitted fact that greater infantile mortality results from abnornal conditions of the oral cavity than from all other diseases combined. The period of first dentition is the most critical period of life. If the development of the tooth is more rapid than the absorption of overlying tissues, local inflammation results; the oral secretions are increased and become abnormal in character; reflex pressure on the nervous and vascular supply augments the trouble, and the entire system becomes involved. Nourishment is refused, the alimentary canal becomes too active, relaxation of the vital forces follows; fever, convulsions, paralysis follow, and death terminates the struggle.

From the anatomy and functions of the trigeminus it often becomes the source of remote lesions—neuralgia, auritis—during the eruption of the first permanent molar. Impacted third molars are the frequent source of intense suffering and serious trouble. Dyspepsia follows the loss of masticatory power, due to diseased or lost teeth. Lowered jaw-vitality invites disease.

Any pathological conditions in the oral cavity causing deficiency in the quantity or quality of the fluids which the food first meets become a hindrance to nutrition and make prompt impression upon the stomach. Females are often anæmic, debilitated, despondent; the physician discovers nothing wrong in any special organ and pronounces "nervous debility," prescribes tonics, change of air, etc. An examination of the oral cavity would too often reveal a seething pit of filth, suppurating sinuses, and carious teeth, contaminating every breath of air taken into the lungs, and vitiating the oral fluids commingling with the food, poisoning it ere it reaches the stomach, causing impaired nutrition, lowering the vital forces and causing the general breaking down in health. Physicians and dentists should co-operate more cordially. The physician should know more of dentistry, the dentist more of medicine. The medical colleges should have chairs devoted to the principles and practice of dentistry, and dental schools should teach more of medicine.

In the discussion of the paper Dr. Younger emphasized especially the disastrous effects of pyorrhœa alveolaris and cited two strongly illustrative cases out of the very many that have fallen under his observation.

Dr. Frank Holland, Atlanta, cited a case in which a lady was under treatment by a prominent surgeon, of Atlanta, for nasal trouble subsequent to a severe attack of la grippe. She had been under his special treatment for six months without the slightest benefit. Visiting Dr. Holland for dental services, he, at once, found that the trouble was due to pus in the antrum from an abscessed central incisor, from traumatic injury, the patient having had a fall some time previous, striking the central incisor and causing the death of the pulp. It had remained in a dormant condition until roused by the attack of la grippe, when an abscess developed which was unobserved and neglected under the nasal specialist's treatment.

Dr. John S. Thompson, Atlanta, cited a case with similar results, but due to an impacted superior third molar. In this case it had been found necessary to remove the second molar and cut out the third molar, which was imbedded in the process with the roots turned up and entangled with the roots of the second molar. Hemorrhage from the nostril followed the removal of the molars and the opening into the antrum was found. With proper treatment the man was soon restored to his field of duty, a healthy man once more.

Dr. Rosser, Atlanta, cited the case of a lady who had been under a physician's care for seven years. She visited his office for dental examination, being brought in supported by her husband on one side, her brother on the other, and accompanied by her daughters. He found her mouth in a dreadful condition, with only three sound teeth—seven broken off and with fistulous openings, pressure with the fingers at almost any point causing pus to exude from the gums. She was, of course, unable to masticate her food, but swallowed it as best she could, with these cesspools continually pouring their contents into her stomach along with the unmasticated food. She was dyspeptic, hysterical, emaciated, and had not known a night's rest for years. She had not been able to sit up all day for seven years, and all the time under a physician's care! In the removal of the useless debris of broken-down teeth it was found necessary to dissect out much necrosed alveolar process. The mouth was put in proper condition, and two months later when a pleasantly, smiling, well-looking lady came in, and addressing him by name, he was quite unable to recognize her until she recalled the circumstances of her previous visit. She said that she felt perfectly restored in health, flesh, appetite and spirits, and had had occasion to call in the physician but once since she had received his dental services. She now enjoys her food which she is able to masticate and which is, apparently, well-digested an assimilated.

Dr. Rosser cited another case in which pus from a suppurating second superior bicuspid had burrowed between the palatal plates, escaping behind the soft palate, being under a physician's treatment for "throat trouble." The sinus was readily traced from the dead tooth, and with proper dental treatment the *throat trouble* disappeared.

Prof. Andrews: Such typical cases as these are of interest to physicians as well as to dental specialists. If others have similar cases it would be well to have them related.

Dr. Gayle, Atlanta: I had a patient whom the physicians had treated in vain for catarrh. She came to me for treatment of an abscessed central incisor. When that was cured the catarrh also disappeared.

In another case the patient, a lady, had been bedridden for five years and was supposed to be about to die from extreme emaciation. She was found to have a severe case of pyorrhœa alveolaris. Both upper and lower teeth were so very loose that they were all removed. Her father, however, would not have teeth made for her as she was about to die.

She did not die, however, and after ten or twelve months had made such progress toward recovery that full upper and lower dentures were inserted, and she eventually recovered her normal health and weight.

Dr. Talbot said that he was much interested in the paper read by Dr. Foster, because, in his lectures in the medical college with which he is connected, he makes a specialty of the relation existing between dentistry and medicine. The late Dr. Allport was influential in the introduction, in the seven medical colleges in Chicago, for Chairs of Dental and Oral Surgery, and they ought to be established in every medical college. It is doubtful if there is a medical college in existence that would not be glad to have this feature introduced. So many pathological conditions of the body are due to the lesions in and about the mouth; very few physicians realize how much general disease is the result of oral conditions—abscessed teeth, pyorrhœa, etc., or the great benefit to the general health to be derived from putting the mouth in a healthy condition. In one case, after the removal of all decayed teeth past saving and all teeth loosened from pyorrhœa and putting the mouth in general good condition, the patient gained thirty-five pounds in weight in three months. Dr. Talbot said that from his position in a medical college he was able to impress upon physicians and students that it was useless to treat patients until the mouth was restored to a healthy condition, and the wonderful effect upon the general system produced by putting the mouth in hygienic condition. He makes a point with students that in diseases of the nasal cavity, fistulous openings of the head, face or neck, the teeth shall, first of all, be thoroughly examined in seeking for *the cause*, because in a large majority of cases *dead teeth* will be found to be the original source of trouble. He endeavors to impress upon physicians the importance of an examination of the teeth and the necessary treatment of the mouth as a preliminary to, and adjunct of, constitutional treatment. Subject passed.

Dr. H. D. Wilson, Bainbridge, Ga., read a paper on the "Amenities of Professional Intercourse," "a poem in prose," which was passed without discussion, the essayist having completely exhausted the subject.

Dr. W. E. Walker, Pass Christian, Miss., read a paper (No. 2), entitled "The Movements of the Mandibular Condyles and Dental Articulation."

This paper, Dr. Walker stated, is a continuation of the line of thought and experiment indicated in a paper read before the Southern Dental Association in 1895, and published in the *Dental Cosmos*, January, 1896, being additional arguments in favor of, and further proof by different methods, of the position taken in the first paper, viz.: That the movement of the condyle, in the function of mastication, or in opening the mouth is not only forward, but *also downward*, in a direction bearing, in each individual a definite relation to the facial line expressed by the angle formed at the intersection of the two lines—the condylo-facial angle and consequently the condylo-occlusal angle, governing the articulation of the teeth. These angles vary very considerably, not only in different individuals but even in the two condyles of most individuals. In the lateral movements of the jaw the condyle on the side from which the jaw is receding does not stand still, or "merely rotate on its axis," as stated by the authorities, but moves upward and backward in a definite ratio to the forward and downward movement of the condyle on the side toward which the jaw is advancing—very slightly in some cases but very considerably in others, and not infrequently in very different ratio in the two condyles of the same individual. Subsequent to the publication of his first paper, the writer having requested the assistance of friends having better opportunities for literary research was referred by Dr. E. C. Kirk to certain similar investigations made under the direction of Prof. Henry P. Bowditch in the physiological laboratory of the Harvard Medical School, of which a report was published in the Boston *Medical and Surgical Journal*, July 4, 1889. He was much gratified to learn that these distinguished investigators, working by widely different methods, had also reached the conclusion to which he had been forced, namely: that "certain errors exist in all descriptions of this articulation given by the eminent anatomists and physiologists."

To further verify the results of his own original methods of investigation and proof, Dr. Walker constructed an apparatus similar to that described as having been in the Harvard investigations, and was gratified to find that by the photographic method his observations were fully corroborated. A light framework being securely attached to the lower jaw, with a bright bead placed directly over the condyle, the line traced by the bead in the movements of the jaw as photographed, shows exactly both the extent and the direction of the movement of the condyle. A series of these photographs accompanied the paper, showing the movement of both the right and left condyle, in opening the mouth to its widest extent, in the lateral movements, as in grinding the food, and in the protrusion of the jaw, to set the teeth on edge, as in biting. Dr. Walker showed also a series of lead-pencil tracings made by the movement of the condyle, a small bit of pencil-lead having been, in this series of experiments, affixed to the framework in place of the bright bead. A piece of stiff paper being held against the face with the edge parallel with the facial line, the movement of the jaw caused the lead-point, placed over the condyle, to trace upon the paper the extent and the direction compared with the facial line, of the movement of the condyle.

A special interest attaches to the relations between the line thus traced by the condyle—in the movements of the mandible—and the facial line, for a certain definite relation exists between the cusps of the teeth and their interlocking, in articulation, and the angle made by the intersection of the facial line by what

Dr. Walker calls "the condyle path," that is, an extension of that portion of the condyle-tracing made in the short movements of mastication, lateral and protrusive, the condylo-facial angle; and also the angle formed by the intersection of the condyle-path and the plane of occlusion (the alveolar plane in edentulous subjects), the former angle varying from a very acute angle in some cases to almost a right angle in others, and in some individuals an equally wide difference between the right and the left condyle angles.

To reproduce the perfect articulation by models of the natural teeth, in the different positions of grinding and biting food, right and left lateral movement, forward protrusion placing the incisors on end, etc., it was found necessary to construct an articulator with an adjustable joint, permitting the reproduction of these varying angles.

On the other hand, to correctly articulate artificial teeth it is necessary to ascertain the angle formed by the condyle-path and the facial line and the condylo-occlusal angle. To accomplish this measurement Dr. Walker has devised an appliance which he calls " Facial Clinometer," by means of which the required angles are measured and registered on degree-gauges, the distance which the two condyles travel simultaneously, forward and downward on one side and upward and backward on the other, being registered in millimeters.

The Articulator ( Walker's Physiological Articulator ) being provided with similar degree and millimeter-gauges, the Articulator is set to correspond with the recorded clinometer-measurements, thus securing the exact reproduction of the movements of the mandible for the case in hand, and consequently the articulation of artificial teeth conformably to those of the lost organs in each individual case. (A photograph of the Clinometer in position illustrates the paper).

A knowledge of the true movements of the condyles and their relation to the articulation of the teeth, is of great importance in many departments of dental work—as in the correction of the articulation of the natural teeth, whether in orthodontia or in the treatment of Riggs' disease (commonly known as pyorrhœa alveolaris); in the shaping of artificial cusps, whether in fillings, crown-work, bridge-work or plate-work, and also in the treatment of diseases of the facial muscles, of the glenoid fossæ, or of plastic adhesions.

In the discussion of the paper Dr. Talbot said, that having made a special study of the jaw for the past twenty years, he had frequently met with very puzzling conditions which he could not understand, but upon which the paper of Dr. Walker threw much light. He considered the paper a very valuable one, being a presentation of scientific investigations based on actual facts, and with deductions of great practical value. He hoped that Dr. Walker would continue that kind of work, reviewing what he has done and verifying it by continued observations. More good can be accomplished by sticking to one point and bringing out all there is in it, than by attempting to cover too broad a field.

Dr. G. V. I. Brown Duluth, Minn., said that the paper was of great interest to him, as he had made a special study of mal-occlusion in connection with pyorrhœa alveolaris, as it is in that direction that we must look for the cure of pyorrhœa, rather than to the use of drugs.

Dr. John S. Thompson spoke of the importance of being able to retain the cusps of artificial teeth, as giving the proper masticating surface for grinding the food. By the old methods of articulation it is very difficult to do this. Dr. Walker is undoubtedly on the right track.

Dr. Andrews spoke of the difficulty of procuring characteristic, individualized, artificial teeth. They are all too much alike and faulty in shape.

Dr. Rosser thought the fault in this matter lies largely with dentists in not educating their patients along this line.

Dr. Walker agreed with this, and said that the S. S. White Company had promised to make some teeth with normal cusps from models he had agreed to furnish, but that he had not yet had time to attend to the matter.

Dr. Andrews hoped he would be able to do this as it was a very important matter.

Dr. Walker: When we are unanimous in the demand, the manufacturers will not refuse the supply; but as long as dentures are made with flat surfaces, mere mashing machines, it will be useless for them to make teeth with normal cusps. The Whites say they did, at one time, make teeth with good cusps, but the dentists complained that they had to grind them off, and preferred the old models. I always demand the longest cusps to be had, as I would rather grind down the cusps a little than have to grind out a sulcus and make cusps.

Subject passed.

Dr. E. S. Talbot read a paper (No. 3) on "Pyorrhea Alveolaris." He described the symptoms and progressive or successive conditions, beginning with simple inflammation of the gums, and ending with exfoliation of the teeth, and reviewed the different theories as to the cause of the disease, which, he said, should more properly be called Riggs' disease, the different names given it, which are mostly descriptive of different symptoms or phases of the disease, not being applicable to all cases.

Dr. Talbot rejects the uric acid theory, as *not proven.* He regards the deposits as secondary in importance, and the gum-tissue as the original seat of the disease, the deposits being due to the attraction of dying tissue for the lime-salts held in solution in the fluids of the body.

Among the causes, Dr. Talbot attributes much to *modern dentistry.* The use of the rubber-dam and of wedges; of the appliances used in the correction of irregularities, protruding fillings or abnormal spaces left between the teeth, crown and bridge-work, artificial teeth--especially ill-fitting plates, and other sources of irritation of the gum-margins with remitting inflammation. A study of the structure and location of the peridental membrane, and the anatomy and physiology of the parts involved, throws much light upon the subject. The disease is a condition of atheromatous degeneration, a softening and liquefaction of the tissues, the gums being inflamed from local or constitutional causes, this extending to the capillaries of the peridental membrane, its vitality is impaired. New connective tissue is formed, causing a thickening of the peridental membrane. Atheromatous patches are formed which become infected with pus germs. The granular *debris* or calcic deposits are a secondary consideration, the inflammatory exudate and pus-formation being primary.

The paper was illustrated by a series of photo-micrographs showing the pathological conditions of the peridental membrane and other tissues in different stages of inflammation and degeneration. The cause being understood, relief is easy if taken in time. In the removal of deposits and other foreign substances great care must be taken not to injure the gum-tissue, which must be stimulated and kept hard and firm. The deposits must be cautiously drawn away from the raw edges of the peridental membrane and the parts thoroughly disinfected; tonics to restore the membrane to a healthy condition are also requisite.

All teeth that are very loose in the sockets should be removed and the space between roots and alveolar process disinfected. To contract the gum tightly about the necks of the teeth and prevent further ingress of foreign matters Dr. Talbot finds the officinal preparation of the tincture of iodin the most effective.

In the discussion of the paper Dr. Younger said that he considers pyorrhœa to be a disease of the pericementum and not of the gums or other tissues. He disagrees with Dr. Talbot in treatment except as to the thorough removal of all deposits. He long ago abandoned the use of iodin except in cases of excessive inflammation. What is wanted is to create an irritation that will excite granulation. That is best accomplished with lactic acid which will prove successful in twenty-four out of twenty-five cases. Dr. Younger described a number of cases successfully treated in the clinics of the Stomatological Club, of San Francisco. If this treatment fails it will be because all deposits have not been removed, or because the pockets have not been first cleansed of blood, serum, etc. The lactic acid is best kept in a little test-tube which can be held over the alcohol flame until liquefied and warmed. If not warm it will cause too much pain. One application, *once for all*, will be all that will be required if the deposits have been thoroughly removed and the pockets properly cleansed. He said: "You may laugh, but try it."

Before applying the lactic acid the surrounding tissues should be protected by coating with glycerine and covered with cotton; then flood the pocket. The lining membrane will be exfoliated, contraction follows and the gum soon clings closely to the root again. Then wait a week. If the point of the syringe can be introduced it is proof that the deposits have not been thoroughly removed, or that the application was not sufficiently thorough to cause perfect exfoliation of the lining membrane, and the treatment must be repeated.

When union is not prompt in cases of implantation the application of lactic acid in the socket will secure perfect union, which, Dr. Younger said, upholds him in his theory of persistent vitality, as there could not otherwise be such perfect reattachment.

He said: "By my method take one tooth at a time and give one, two, three or four hours, if necessary, to the removal of deposits. The next day take another tooth in the same way. If there are three contiguous teeth to be treated, clean the central tooth and the adjacent sides of each of the adjoining teeth. The next day finish the outer sides but do not disturb the central parts. The treatment is very simple but it *must be thorough*, and be very particular not to do any washing out after applying the lactic acid. Flood the pockets and leave it there. As a subsequent wash chlorate of potash, as strong as can be borne, will be found very soothing."

Dr. Talbot: You mean, I suppose, that there will be contraction of the gum-tissue around the root of the tooth—you do not mean adhesion?

Dr. Younger: I mean *absolute union.* If there is not union there is no cure. After my treatment the conditions are absolutely the same as where there has been no pyorrhœa, or as before the attack.

Dr. B. H. Catching: I have never found any instruments that suit me. Are there any *Younger* instruments? If you have them and will publish the illustrations I would be glad to get them.

Dr. Younger: My fingers are very sensitive and the contact with steel instruments is unpleasant to me. I coat the handles of my instruments with sealing wax, using different colors for different instruments—as red, blue, purple, black, white, etc., so that I know the point by the color of the wax on the handle. I prefer a large handle—from one-half to three-quarters of an inch through. With such a handle you get a power and force that you can not get with instruments with small handles of steel or iron.

Dr. R. B. Adair, Gainesville, Ga., said that he had been treating pyorrhœa for twelve or fifteen years in a rural district where there was scarcely a patient but who had more or less of it. He considers the disease just as amenable to treatment as anything else in the line of dental practice. Cases that he treated and *cured* twelve or fifteen years ago have remained perfectly sound and well to this day. He has recently seen one of the first cases that he treated and which he reported before the Georgia State Society, in which there had been ten deep pockets. The patient came in again recently for fillings in two teeth. The pyorrhœa has remained cured and the gums are sound to-day.

While he is grateful to Dr. Talbot for many points brought out in the paper he takes issue with him as to the initial seat of the disease, having seen cases where the deposits were to be found only near the apex of the root without any external opening—an external fistula present but with the margins of the membrane firmly attached. He was also not so particular about not wounding the peridental membrane. He uses Allport's scalers, which are thin and flexible, and go right to the bottom of the pocket and tear out everything, scraping away the necrosed edges of bone, getting new tissue.

He would have hesitated to use lactic acid as it breaks down bone-tissue very rapidly so that it can be bent double after twenty minutes immersion. But he makes a point of never letting scientific theories interfere with clinical observation, and with such high indorsement as that of Dr. Younger he intends to try it at the first opportunity.

Dr R. R. Andrews (to Dr. Younger). Is it a fact that where there is slight decalcification there is more ready adhesion as claimed by recent French observers?

Dr. Younger replied that while the theory referred to was interesting *as a theory*, practically he thought it would prove a failure—that is, the partial decalcification of teeth to be implanted.

Dr. G. V. I. Brown, Duluth, Minn.: Considers the esentials in the treatment of pyrrhœa to be—

*First*, The thorough removal of all deposits, using an acid if necessary, to decalcify the inaccessible portions.

*Second*, Getting the gum to grow up around the tooth again.

As to the causes of the disease he does not think that the rheumatic diathesis theory has had sufficient confirmation to be accepted. It may, ar d often does, exist incidentally, but not as a *cause*. He considers that mal-occlusion is the most frequent cause. As the result of mal-occlusion the surrounding tissues are irritated and become hyperæmic, the tooth is elongated and irritation is increased, resulting in one of two things: either the *formation* of something or the *destruction* of something. He objects to the use of the word *cure*, as many cases that are *apparently cured* do not *stay* cured.

Dr. Walker does not think Dr. Talbot's argument against the lactic acid theory a sound one—viz.: That *all* the teeth would be equally affected. All the toes and finger-joints are not affected in gouty subjects; it may be only one big toe-joint or only one little finger, and in the same way it may be only one or two teeth. The evidences of this constitutional dyscrasy are very varied. In one it may affect the finger-joints, in another the bronchi, in another it takes the form of skin disease. In some it manifests itself in one form and in others very differently. He said: "In all the cases that have fallen under my observation since Dr. Pierce called attention to this view—I was too young when Dr. Pierce's paper was published for it to attract my attention—I have had but one case in which there were not other symptoms of lithemia. This was a young lady of twenty-four or five who was suffering from Rigg's disease due to faulty articulation, the upper lateral having been pushed out of position by faulty occlusion through loss of the posterior teeth. The vital tissues did not follow up the tooth. There were no deposits, but there was a pus-pocket. I have been seeing this case for a year and a half. In many cases where there is no trace of uric acid in the urine, after failing in other methods of treatment the lithic acid treatment will effect a cure. Another point: By certain modes of living, going on a debauched indulgence in highly nitrogenous food, etc., a person may bring on such a condition of blood as to cause deposits upon the teeth; by change of habits, and possibly of climate, all trace of uric acid may subsequently be eliminated from the blood and from the urine, so that when, at a later period, treatment is had for Riggs' disease resulting from the deposits above mentioned, examination would fail to reveal any constitutional lithemia as cause for the deposits. But these will remain unchanged upon the teeth unless, mechanically removed, long after all traces of systemic lithemia may have been eradicated. When I undertake the treatment of a case I tell the patient that I can cure him if he will help me, but I must have his co-operation in diet and habits of living as well as in the care of the mouth prescribed. Constitutional treatment I find, from my experience in the treatment of the disease, to be essential in lithemic cases, but constitutional treatment without the removal of the deposits will not cure the disease. I remove the deposits with instruments as perfectly as possible. If inflammation and pus-formation persist I go for more deposit. If instruments do not find it I use trichlorocetic acid to dissolve those minute particles that defy instrumentation. I recall a case that I first had under treatment about four years ago, a lady. There were heavy deposits of serumal calculus, with deep pockets reaching to the bifurcation of the roots—both upper and lower teeth—the gums were so inflamed that they were purple. I am satisfied that she would have lost her teeth within six months. There were no salivary deposits. I removed the deposits; requiring several sittings, and treated as usual. At the end of a month I found a little calculus here and there, but at the end of three months, and then six months, and then eight months more, there were no more deposits, no pus, and the gums were as healthy as previous to the attack.

Lithemic patients do not stand a cold climate well and Pass Christian is a favorite resort for this class of sufferers, so that I have occasion to see a great deal of it and to treat pyorrhœa for them. Cold weather favors the accumulation of lithates in the system, from arrest of perspiration, and they instinctively seek a warmer climate in winter. That is, perhaps, why so many of those who come to me for treatment of Riggs' disease are lithemic sufferers. I had a patient last winter, a lady from Montana. She stayed south much longer than she had intended to do because I said I could cure her, her own dentist having said it was impossible to save her teeth.

It required five sittings for the removal of the deposits. She had other marked lithemic symptoms, and in addition to my special treatment for pyorrhœa, or Riggs' disease, I put her on tartar lithemic to her very great benefit.

I will ask Dr. Talbot for an explanation of his use of the term *neurotics.* I do not find it in the dictionaries. What does he mean when he says that, "the children of neurotics are generally either neurotics or degenerates?"

Dr. E. R. Carpenter, Chicago: The most aggravated cases in my experience are those in which we are unable to find any deposits. The anterior teeth are very loose, with a relaxed condition of the gum-tissue. The deposits are a result and not the cause; pyorrhœa goes in advance of the deposits. From the relaxed condition of the tissues anything that might tend to deposit will be washed out by the buccal secretions and the teeth be found clean and nice but with the gum hanging off loose. Where there are deposits we are apt to leave a very thin layer, really beveling off the edges of the deposit. The officinal dilute sulphuric acid assists greatly in the removal, and does not act upon the tooth-tissue as it does upon the deposit. I have never expected to secure adhesion of the gum; the best I hope for is to get control of conditions, but I have to watch my cases and have these return for examination. I have never had the results in treatment that Dr. Younger claims to get from the use of lactic acid, though I have not used that agent.

Dr. Walker: When I speak of cure I do not mean to say that there is an absolute certainty that it will never return. I use the word cure in the sense that the physician does when he cures a lithemic skin disease or lithemic bronchitis. But if instructions are followed the disease can be cured.

Dr. B. H. Catching: There are very few men who are entitled to have an opinion on such subjects. It is only those who have made scientific investigations that are entitled to an opinion. Mere clinical experience is not sufficient for a writing of this character. Dr. W. D. Miller, for instance, has the right to express an opinion as to the cause of caries, because of the scientific investigations he has made. I agree that we ought to call it Riggs' disease, for Dr. Riggs was the first one to really place it before the profession. I am glad to hear it called Riggs' disease, and I shall stick to that name. All cases that are called Riggs' disease are not entitled to that name.

Dr. Walker: I will ask Dr. Catching to tell us what Riggs' disease is?

Dr. Catching: It will take some one else than Dr. Catching to tell you that!

Dr. Andrews: These photographs of Dr. Talbot's are very fine. These dark spots mark newly formed-tissue which takes stain easily and shows very clearly the differentiation. I have not made a study of these diseased tissues myself, but from my general knowledge of the subject I judge that the photographs are most excellent.

Dr. Talbot (to close): The main point still is that we must find *the cause* as Dr. Miller has found the cause of caries. Until we know the cause our treatment must be at random. These photographs show that even after the gums have contracted and there can be no further ingress of pus germs, yet deep down, three-quarters of an inch below the suppuration line, there is still an invasion of inflammatory tissue, deep-seated disease in the peridental membrane, and we don't know when it is cured. The most we can do is to prevent further intrusion of foreign substances.

As to the question, what is a neurotic? The subject of degeneracy has lately come prominently to the front, and there is a difference of opinion as to the distinction between neurotics and degenerates. I might attempt to express it by saying that in the neurotic the nervous system is unstable. The degenerate goes back from a normal to a lower form—but they are really one and the same thing. A paper to appear in the next issue of the *International Journal* will give a fair idea of the meaning of the terms.

Dr. T. P. Hinman exhibited the models and photographs and explained his methods in modeling in a very remarkable case of restoration of lost tissues from the eyebrow down to, and including, the upper lip and maxilla, the restoration being effected by a hollow aluminum piece carrying the teeth of one-half of the maxilla, the upper portion forming the lower border of the socket of the lost eyeball.

Dr. S. W. Foster, who had followed the case during the operations, expressed himself as delighted with the success achieved. The articulation obtained was something wonderful, while at a distance of twenty feet the artificial character of the restored portions of the face both bony and soft tissues having been destroyed from nose to ear and from eyelid to lower lip.

Dr. Talbot read a paper by Dr. W. X. Sudduth on "Modern Methods of Treating the Maxillary and other Sinuses of the Cranium."

He said that empyema of the antrum, in a large proportion of cases, is due to infection from dental abscesses, or to extension of disease from nasal cavity. The most common causes of obstruction of the natural outlets are polypi and decomposed secretions. As the result of suppuration within the cavity of the antrum, the lining membrane becomes thickened and spongy and the caliber of the cavity is reduced in consequence. Neuralgias follow the course of the nerve supply causing sympathetic affections of the eye, generally unilateral. When the natural outlets are obstructed matter accumulates with consequent pressure. Necrosis of the walls of the antrum causes a discharge into the nasal cavity, or through absorption of the orbital plate matter infiltrates into the tissues about the eye. Pus may discharge into the throat interfering with the general health, causing dyspepsia and poisoning the whole system.

The methods of establishing differential diagnosis between the nasal and dental sources were described minutely.

Trans-illumination has failed in the hands of the most expert specialists in actual practice.

In treatment he would prefer to treat through the septum rather than sacrifice a good tooth to treat from within the oral cavity; or, would trephine in the region of the canine fossa if the patient objects to a tube exposed in the face. If the latter method is selected, a trephine and flexible rubber catheter of the same caliber should be selected and placed in an antiseptic solution, the mouth being rinsed with borated soda or listerine. The first dressing may be introduced through the opening made by the trephine. A band should be cemented to the nearest tooth with a piping cemented to the band, which, to secure the tube, the rubber-tube should be three or three and a half inches long, and one half or three-fourths of an inch passed into the antrum, the free end being kept plugged with a soft wooden pin to prevent the entrance of saliva or air—sources of renewed infection. A bi-borate of soda solution is to be introduced through the tube into the antrum and allowed to flow out into the nasal cavity, carrying with it the pus from the antrum, the patient inclining the head forward, a freer opening being made into the nasal cavity if necessary. After the cavity is thus cleaned, 3 percent pyrozone should be introduced to disintegrate any remaining pus, completing the dressing with a bland antiseptic, as listerine, left in the cavity. This, and other cavities of the cranium, can be easily washed out in this manner by the patient, or by friends or family, and should be repeated two or three times a week. If any odor is perceptible after the first treatment, permanganate of of potash may be substituted for the listerine, for a few days. Laterine, when the flow of pus is materially diminished, stimulating antiseptics may be used, as sozo-iodol and its compounds. In acute cases, sozo-iodol and bismuth; in sub-acute cases, sozo-iodol and mercury; in chronic cases, sozo-iodol and zinc. All solutions should be used warm.

In the discussion of this paper the Chairman spoke of the very great success in trans-illumination obtained by Dr. Cobb, of Boston, and attributes failure in the use of too small a lamp. Dr. C. uses 8-candle power, putting on and turning off the current as it gets too hot. In fifteen cases he showed very clearly the condition, the diseased antrum contrasting strangely with the other side.

Dr. G. V. I. Brown does not quite agree with Dr. Sudduth in regard to differential diagnosis, and does not like a tube projecting so far into the antrum, as the remedial agents can not wash all points in the antrum when it is thus introduced. Neither would he instruct the patient to leave any fluid in the antrum as its presence is liable to cause neuralgias. Good drainage is very essential.

Dr. Talbot questions whether any large proportion of diseased antri is due to diseased teeth. Other causes are: Nasal troubles, adenoid vegetation, mouth breathing irritation of the nasal membrane extending into the antrum, the severe storms of the Northwest, blizzards, etc., causing acute inflammation of the mucous membrane. The proper place for trephining is between the roots of the second bicuspid and the first permanent molar, but these cavities are frequently deformed, especially in degenerates, and are seldom found as portrayed by Gray. In some cases the antrum is entirely obliterated on one side, while the other side may be twice the normal size. Sometimes the nasal cavity extends very far to one side. He has seen two cases where the dentist had extracted the tooth and drilled the alveolus, penetrating the floor of the nose instead of entering the antrum. At that time he thought it was due to the ignorance of the operator, but he now recognizes that it was because of a not uncommon malformation. Often there are septæ reaching across the antrum forming two cavities, so that any treatment reaching one portion would fail to benefit the other. He does not think the antrum can be drained by the middle meatus of the nose, which is half way up the side of the wall. He doubts if even pyrozone would blow it all out. The suggestion of plugging the other end of the tube is a good one, as there is no part of the body so liable to communicate infection as the mouth.

Dr. Brown: And yet, in no part of the body will a wound heal so readily as in the mouth. We can make incisions, cut out portions, and the wound will heal without trouble, as it would not do in the hand or the arm. So long as the wound is open, and the secretions not pent up, we will not have infection in the oral cavity from the oral fluids.

The Chair: A child instinctively puts its mouth to a wound to cure it; it is Nature's great healer.

Dr. Talbot: And animals also do the same thing.

Dr. Brown: When a tooth is extracted the vessels of the socket are all open to infection, and yet we seldom have any trouble. Granulation proceeds from the bottom, and it gets well without any attention. Nowhere else is it done with similar ease.

Dr. Walker: I have often noted, with surprise, this ready healing in the mouth. If the gum is scarified extensively, and the hand scratched even very slightly with the same instrument, the mouth will heal promptly but you may possibly lose the hand or the finger.

Dr. G. V. I. Brown, Duluth, Minn., read a paper on "The Disinfection of the Mouth as a Potent Factor in the Treatment of La Grippe." If physicians fully understood the necessity of disinfection of the mouth, it would be made as much a part of preliminary treatment as the administration of a cathartic, and this would also serve to bring dental and medical practice into correlation.

What is wanted is a plain statement of facts, of observation, and experience in relation to particular diseases. This is especially the case in regard to influenza (la grippe), a disease, of which the real nature is shrouded in mystery, the etiology comparatively unknown, and of which the complications and sequellæ form the most formidable part, due to secondary infection.

The diagnosis is not difficult; the treatment is mostly symptomatic; the prognosis is favorable except in cases of heart or pulmonary trouble, or with chronic-necrosed patients.

Discharges of pus in the oral cavity, as from abscessed teeth, carious and necrosed jaws, pyorrhœa alveolaris, aggravate the symptoms, with danger of direct infection from the resorption of the waste products of toxic poisoning, and the passage of pus-germs through the alimentary canal ready to incite inflammation and catarrhal processes wherever lowered vitality offers the least resistance.

Over 90 percent of cases will be found to have some pathological condition, and yet medical literature says nothing about direct medication for the disinfection of the sulcus a portal of infection.

Not that la grippe is purely and simply the result of oral infection, but the secretions of the mouth offer the best culture-medium for its specific organism, and much benefit would be derived and serious complications avoided by the prompt, continued and thorough disinfection of the mouth, not only by sterilizing the secretions, but through the treatment and removal of diseased conditions.

What is true of la grippe is equally true of many other diseases, and far-reaching benefit would result from establishing the practice of mouth-disinfection as a part of the general treatment of disease.

This paper was highly commended, but passed without discussion owing to limited time.

A paper from Dr. H. W. Gillette on "Cataphoresis" was next read, and the Section then adjourned till the next annual meeting, in Philadelphia, the second Tuesday in June, 1897.

